# Genetic study on candidates for oocyte donation

**DOI:** 10.5935/1518-0557.20240087

**Published:** 2025

**Authors:** Sara Araújo, Ana Paula Neto, Maria João Pinho, Sofia Dória, Alberto Barros, Filipa Carvalho

**Affiliations:** 1 Genetics Unit, Department of Pathology, University of Porto, Alameda Professor Hernâni Monteiro, 4200-319, Porto, Portugal; 2 Centro de Genética da Reprodução Prof. Alberto Barros, Av. do Bessa 240, 4100-012 Porto, Portugal

**Keywords:** carrier screening, oocyte donation, gamete donors, expanded carrier screening, case series

## Abstract

**Objective:**

There is a rising demand for assisted reproductive medicine, including sperm, oocyte and embryo donation. Besides medical and legal considerations, genetic testing, including carrier screening for multiple autosomal and X-linked recessive disorders plays an essential role in evaluating hereditary risk among donors and therefore exclude them from the donation process.

**Methods:**

A retrospective study was conducted on oocyte donors from a private clinic of assisted reproduction who underwent genetic testing between June 2014 and September 2023. Pre and post-test procedures were performed at the private clinic while karyotyping and carrier screening for Cystic Fibrosis, Fragile X syndrome and Spinal Muscular Atrophy were performed at the Genetic Unit of Faculty of Medicine, University of Porto.

**Results:**

Among 581 donors, 81 women were excluded from the donation process since 5/563 had an alteration in karyotype, 57/581 were carriers of a Cystic Fibrosis Transmembrane conductance Regulator pathogenic variant or had a 5T allele, 11/394 had Survival of Motor Neuron 1 deletion and 8/426 had an intermediate or premutation allele in Fragile X Messenger Ribonucleoprotein gene. While recommendations from fertility societies advocate for comprehensive screening, opinions differ on the mandatory implementation of expanded carrier screening.

**Conclusions:**

In conclusion, the genetic tests and the pre and post-test counseling is imperative to optimize reproductive outcomes in the oocyte donation process.

## INTRODUCTION

The number of people wishing to have children, and who resort to sperm, oocyte, and embryo donation has significantly increased over the past decades (Portal CNPMA, 2024). This process is closely guided by medical, legal and genetic professionals and follows specific guidelines to improve safety and outcomes (Practice Committee of the American Society for Reproductive Medicine and the Practice Committee for the Society for Assisted Reproductive Technology, 2021).

Therefore, in addition to collecting the donor’s medical history, which gives information about possible familial hereditary diseases, medical backgrounds, mental health, risky sexual behaviors, among others, it is also necessary to carry out genetic tests. These tests can provide valuable information about specific genetic diseases, such as Cystic Fibrosis (CF), Spinal Muscular Atrophy (SMA), Fragile X syndrome, among others (Practice Committee of the American Society for Reproductive Medicine and the Practice Committee for the Society for Assisted Reproductive Technology, 2021; Soares *et al.*, 2023).

Carrier screening (CS) is understood as a type of genetic test that can determine if an individual is carrying a pathogenic variant for a certain recessive condition or a X-linked disease and it is offered to those who do not have a known family history of genetic disorders. This type of test focuses on a specific set of diseases while extended carrier screening (ECS) tests are performed for a wider range of variants and genes associated with several genetic disorders (Payne *et al*., 2021; Practice Committee of the American Society for Reproductive Medicine and the Practice Committee for the Society for Assisted Reproductive Technology, 2021).

In Portugal, oocyte donation is a legal practice regulated by Law no. 32/2006. To become an oocyte donor, women must meet certain requirements, such as being between 18 and 34 years old, being healthy, not having sexually transmitted, genetic, or other considered serious diseases, as well as not having a family history of genetic diseases (Portal CNPMA, 2024).

The American Society for Reproductive Medicine and the Society for Assisted Reproductive Technology recommends screening for pathogenic variants associated with the Cystic Fibrosis Transmembrane conductance Regulator (*CFTR*) and Survival of Motor Neuron 1 (*SMN1*) genes. Karyotyping is considered optional. An update of these guidelines also includes screening for hemoglobinopathies for all candidates, as well as testing for the CGG expansion of the Fragile X Messenger Ribonucleoprotein 1 (*FMR1*) gene, responsible for the Fragile X syndrome, even in the absence of family history (Mertes *et al*., 2018; Practice Committee of the American Society for Reproductive Medicine and the Practice Committee for the Society for Assisted Reproductive Technology, 2021).

This study aims to analyze the results of the genetic study carried out on a series of oocyte donors from a private clinic of assisted reproduction.

## MATERIAL AND METHODS

### Study design and participants

This retrospective case series study was conducted using the database of Genetics Unit, Department of Pathology, Faculty of Medicine, University of Porto. The data presented was collected from potential oocyte donors of Centro de Genética da Reprodução Prof. Alberto Barros between June 2014 and September 2023. Standard procedures not related to genetic testing (clinical history, informed consent, collection and preservation of oocytes and genetic counseling) were carried out in the private clinic. Carrier screening for *CFTR, SMN1* and *FMR1* genes and karyotype were performed at Genetics Unit, Department of Pathology of Faculty of Medicine, University of Porto.

### Oocyte donors

The initial stages of the oocyte donation process took place at the private clinic, where prerequisites were verified. A personal and family history of hereditary diseases, sexually transmitted diseases and mental health was also collected. In addition, the stages of the donation process, the risks and benefits and the associated legal conditions were explained. After checking all the necessary conditions, the next step was to perform specific genetic tests. A blood sample was sent to the Genetics Unit, Department of Pathology, Faculty of Medicine, University of Porto for karyotyping and carrier screening.

### Karyotype and carrier screening

For karyotyping, lymphocyte culture was performed with high resolution metaphases being stained using the GTL-banding technique (G-bands by Trypsin using Leishman).

To screen for *CFTR* gene (OMIM * 602421; 7q31.2), the Elucigene^®^ CF-EU2v1 kit and Elucigene^®^ CF Iberian Panel were used, which identifies the 50 most common pathogenic variants in the European population and 12 pathogenic variants most frequently found in the Portuguese and Spanish population, respectively. In addition, the first kit also analyzes the Poly thymidine (PolyT) polymorphism in intron 8 and measures the adjacent TG repeat.

To screen the copy number of the *SMN1* (OMIM * 600354; 5q13.2), associated with SMA, the SALSA® MLPA^®^ Probemix P060-B2 SMA carrier was used followed by capillary electrophoresis.

Finally, for Fragile X syndrome, an *in house* Polymerase Chain Reaction protocol was performed to amplify the CGG repeats in the *FMR1* gene (OMIM * 309550; Xq27.3), using the following components: 7 µL of Enhancer System (Invitrogen^®^) (Life Technologies), 6.4µL of water, 2 µL of Buffer, 2µL of dNTPs (10 mM) (Invitrogen^®^), 0.6µL of MgSO_4_, 0.4 µL of FMR1-F Primer, 0.4 µL of FMR1-R Primer and 0.2µL of Taq DNA Polymerase, recombinant (5 U/µL) (Thermo Scientific^®^) (ThermoFisher). The threshold from which Fragile X syndrome is caused is a CGG repeat count of 200 or more. This is defined as a full mutation. The premutation range, which indicates a carrier status, spans from 55 to 200 repeats, and the normal range is between 6 and 44 repeats. CGG repeats from 45 to 54 is known as the grey zone and it has the potential to progress to a premutation when transmitted to the offspring (Metcalfe, 2012; Ontario Health, 2023).

## RESULTS

A total of 581 oocyte donors were included in the study. The mean age of oocyte donors was 27 (ranging from 18 to 34 years). Not all donors performed all the genetic tests. Usually, *CFTR* screening is the first test to be performed in parallel with the karyotype. If an alteration is detected no further tests are done. From June 2014 to September 2023, 563 karyotypes were performed and 581, 426 and 394 carrier screening tests for *CFTR, FMR1* and *SMN1* were done, respectively.

### Karyotype

Of the 563 karyotypes performed, 5 (0.9%) showed a chromosomal alteration: a Robertsonian translocation between chromosomes 14 and 21 (45,XX,der(14;21)(q10;q10)); a monosomy X mosaicism (45,X[3]/46,XX[27]); a reciprocal balanced translocation between chromosomes 7 and 17 (46,XX,t(7;17) (q31.31;p13.1)); a pericentric inversion, involving chromosome 21 (46,XX,inv(21)(p11.2q21.2)); and a fragile site at 9p21.1 (46,XX,fra(9)(p21.1)).

These five women were excluded from the oocyte donation process.

### *CFTR* gene

Of the 581 tests carried out for *CFTR* gene, 10 donors (1.7%) showed at least one heterozygous pathogenic variant. Those pathogenic variants and their allelic frequency are listed in Table 1, with p.Phe508del variant being the most frequent one in accordance with the published data for Caucasian populations (Bobadilla *et al*., 2002).

**Table 1:** Pathogenic *CFTR* variants and respective allelic frequency in the studied oocyte donors.

Pathogenic variant	Allelic frequency n (%)
c.1521 1523delCTT p.Phe508del	6 (0.5%)
c.1624G>T p.Gly542X	2 (0.2%)
c.350G>A p.Arg117His	1 (0.1%)
c.3909C>G p.Asn1303Lys	1 (0.1%)

In addition to the screening for the 62 pathogenic variants in the *CFTR* gene, it was also analyzed the PolyT sequence. The polymorphic thymidine sequence, which is located between intron 8 and exon 9, affects the splicing efficiency of exon 9 and influences transcription of the *CFTR* gene. Five, seven or nine thymidine residues can be detected, referred as 5T, 7T or 9T, respectively. If the 5T allele is identified, it leads to the skipping of exon 9 in transcripts, resulting in the production of a non-functional protein (Tabaripour *et al*., 2012).

In the present series, 47 donors (8.1%) had at least one 5T allele, either in homozygosity (n=3) or in heterozygosity with the 7T allele (n=40) or with the 9T allele (n=4). These results are detailed in Table 2. The allelic frequency of the 5T allele was 4.3%.

**Table 2:** Polymorphic thymidine sequence genotype and respective frequency in the studied oocyte donors.

PolyT sequence genotype	Frequency n (%)
7-7	401 (69.0%)
7-9	125 (21.5%)
5-7	40 (6.9%)
9-9	8 (1.4%)
5-9	4 (0.7%)
5-5	3 (0.5%)

Therefore, after the *CFTR* gene analysis, a total of 57 women (9.8%) were excluded from the oocyte donation process.

### *FMR1* gene

**Figure 1:**
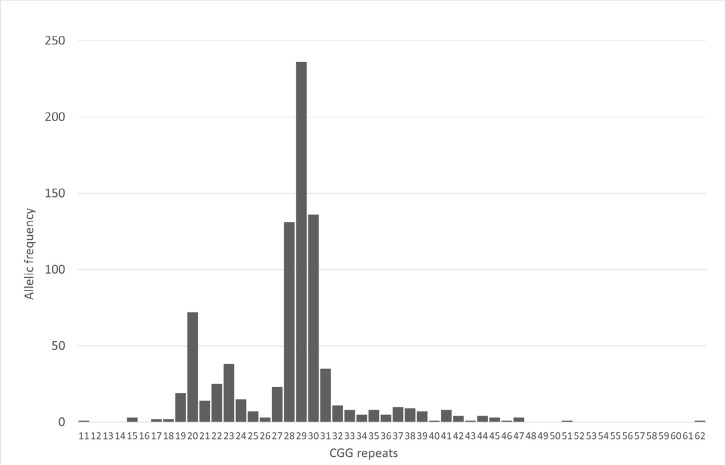
Distribution of the CGG repeats in the studied oocyte donors.

From the 426 tests carried out for the *FMR1* gene, 7 oocyte donors (1.6%) had intermediate CGG repeat i.e. between 45 and 55 repeats and 1 (0.2%) had a premutation of 62 CGG repeats. These eight women were also excluded from the oocyte donation process. The distribution of the CGG repeats in this series of donors is represented in Figure 1, being the allele with 29 CGG repeats the most frequent one.

### *SMN1* gene

From the 394 tests carried out for the *SMN1* gene, 11 donors (2.8%) had only one copy of the *SMN1* gene (Table 3).

**Table 3:** Number of copies of *SMN1* gene and respective frequency in the studied oocyte donors.

SMN1 gene Number of copies	Frequency n (%)
1	11 (2.8%)
2	337 (85.5%)
3	44 (11.2%)
4	2 (0.5%)

All classes of SMA are principally caused by an alteration in the *SMN1* gene. This is the one responsible for producing the functional survival motor neuron protein (Ontario Health, 2023; Yao & Goetzinger, 2016; Zhang *et al*., 2020).

These eleven women were excluded from the oocyte donation process.

Summing-up, after the screening of the three different genes, and including the karyotype analyses, a total of 81 out of 581 (13.9%) donor candidates had an alteration and thereafter were excluded from the donation process.

## DISCUSSION

In the present study the frequency of chromosomal abnormalities in oocyte donors was 0.9% (5/563). Regarding the *CFTR* gene, the frequency of alterations was 9.8% (57/581), 10 harboring pathogenic variants and 47 carrying the 5T allele. One out of 426 (0.2%) exhibited a premutation in the *FMR1* gene, 1.6% (7/426) had alleles within the grey zone of CGG repeats, and 2.8% (11/394) were carriers for SMA. Overall, we thus detected an abnormal chromosomal and genic result in 13.9% of the female donors. It should be taken in consideration that these values are representative of this specific series and may not be extrapolated to other populations with different ancestry.

The comparisons of these results with the literature are depicted in Table 4, showing relatively similar frequency of genetic alterations in this series compared with other reports, and represent a larger series when compared to the other Portuguese study (Soares *et al*., 2023).

**Table 4:** Comparative results of the frequency of alterations in different studies.

Study	Study design	Karyotype alterations	*CFTR* variants	*SMN1* deletion carriers	*FMR1-* premutation
This study	581 female gamete donors	0.9%	1.7%^*^ 8.1%†	2.8%	0.2%
Soares *et al.,* 2023	63 female and 34 male gamete donors	1.03%	5.2%^*^	2.1%	1.6%
Payne *et al.,* 2021	136 female gamete donors		4.4%^*^	0%	1.5%
Zhang *et al.,* 2020	13069 pregnant women			1.77%	
Archibald *et al.,* 2018	12000 individuals		2.9%^*^	2.0%	0.3%
Urbina *et al.,* 2017	127 females and 16 males		4.2%^*^	1.4%	0%
Ben-Shachar *et al.,*^2011^	6394 individuals			2.5%	

* Values for the pathogenic *CFTR* variants;

ⴕ Value for the PolyT 5T allele.

As determined by the reproductive genetics’ clinic, a target approach was performed with karyotyping and carrier screening tests for CF, Fragile X syndrome and SMA.

The prevalence of those diseases plays a fundamental role in their selection for carrier screening. Alpha and beta-thalassemia, sickle cell anemia, hemophilia, CF, TaySachs disease and Fragile X syndrome are identified as the most common recessive or X-linked genetic disorders (Ontario Health, 2023). SMA ranks as the second, after CF, most prevalent, life-shortening autosomal recessive disorder, being an important cause of infant death (BenShachar *et al*., 2011; Sugarman *et al*., 2012). Fragile X syndrome represents the most prevalent inherited condition that causes intellectual disabilities and autism (Ontario Health, 2023).

Regarding carrier screening in gamete donation, various sources and societies in the field of fertility and reproduction offer recommendations and guidelines that outline the diseases to be tested, indicating which are obligatory, recommended, or optional (Capalbo *et al*., 2022). It is essential to include the most common autosomal recessive diseases and adjust them to the population based on ethnicity and country of origin. Most of those societies recognize the significance of pre-test genetic counseling, but they have different approaches when it comes to genetic carrier screening tests (Capalbo *et al*., 2022).

The European Society of Human Reproduction and Embryology (ESHRE) recommends screening for the most prevalent autosomal recessive disorders but mention that the study of the CGG expansion and the routine karyotyping should be optional (Dondorp *et al*., 2014). The American Society for Reproductive Medicine (ASMR) and the Society for Assisted Reproductive Technology (SART) recommends carrier screening for CF, SMA and hemoglobinopathies in all candidates. They consider the karyotyping optional and state that screening for CGG repeats in *FMR1* gene should be weighted in female donors. Moreover, they state that additional CS should be adapted on the donor’s ancestry (Practice Committee of the American Society for Reproductive Medicine and Practice Committee of the Society for Assisted Reproductive Technology, 2022). The American College of Medical Genetics (ACMG) recommends carrier screening for SMA and CF in all donors (Zhang *et al*., 2020). The American College of Obstetricians and Gynecologists (ACOG) recommends carrier screening for SMA, CF and hemoglobinopathies in all donors and, in addition provides guidelines regarding the characteristics that should be taken into consideration of the disease screened (Capalbo *et al*., 2022; Grody *et al*., 2013). Both also recommend a list of eight diseases to screen the carrier status if the individual has an Ashkenazi Jewish heritage (Westemeyer *et al*., 2020). The Royal Australian and New Zealand College of Obstetricians and Gynecologists (RANZCOG) recommends carrier screening for thalassemia, CF, SMA and Fragile X syndrome to all population and additional diseases in specific populations (Capalbo *et al*., 2022).

With the advances of genetics, it is now feasible to screen a broader range of disorders and include a growing variety of different pathogenic variants, with increased accuracy, rapid processing, and reduced expenses (Dondorp *et al*., 2014). Similar to the conventional carrier screening, ECS, also focuses on identifying autosomal or X-linked recessive diseases that predominantly impact newborns and infants leading to cognitive impairment and physical disabilities (Lazarin & Haque, 2016; Yao & Goetzinger, 2016).

There are studies referring the implementation of ECS both in gamete donors and the general population. With the use of ECS it is expected an increase in the frequency of identified alterations. Therefore, the wider the range of genes and diseases tested by ECS, the greater the number of carriers will be identified and, as most guidelines advise for the exclusion of donors if they are carriers of a recessive disorder, the number of donors available will reduce (Payne *et al*., 2021).

In a study, using an ECS panel that screens 46 diseases, it was detected that 17.6% of 883 gamete donors were carriers of at least one alteration (Payne *et al.*, 2021). Another study, using an ECS panel that screens 314 diseases and 2719 pathogenic variants, showed carrier frequencies of 41% in 143 donors (Urbina *et al*., 2017). Using an ECS panel that screens 20 diseases, other authors found a 10.4% carrier frequency in a general population of 3877 individuals, and 9.3% in 1212 gamete donors (67 males and 1145 females) (Capalbo *et al*., 2021). Another study performed in a general population, screening for 100 diseases by an ECS panel, showed that 35% of the individuals were carriers of at least one pathogenic alteration (Yao & Goetzinger, 2016).

Due to its increased availability, laboratories started offering ECS. The current ECS panels exhibit significant heterogeneity in terms of panel size, spanning from 41 to 1556 diseases screened (Westemeyer *et al*., 2020). However, there is no professional guidance on which genes and pathogenic variants to include, and this decision is not straightforward (Grody *et al*., 2013). Despite that, the American College of Obstetricians and Gynecologists (ACOG) suggests that a practical requirement to include a disease is a carrier frequency ≥1:100, which corresponds to an incidence of 1:40000. The goal is to, while recognizing more carriers of prevalent diseases, reduce the stress caused by a positive result for a rare disease (Ontario Health, 2023).

There is a discussion about considering ECS as part of the routine tests for gamete donors, but also in a preconception and prenatal scenario (Mertes *et al*., 2018). When using ECS, there is a capacity to identify more at-risk individuals and couples, allowing the assessment of a genetic risk and reproductive planning to prevent the transmission of detectable hereditary diseases to future generations (Ontario Health, 2023). Comparing to generic CS, the low cost of ECS, achieved through advances in DNA sequencing, further supports its utilization (Urbina *et al*., 2017). Additionally, some individuals view ECS as an additional advantage during gamete donation, as it provides valuable information for the donor own reproductive choices (Pennings, 2020).

On the other hand, concerns have been raised regarding the use of ECS. With expanded gene and disease panels, the number of oocyte and sperm donors will tend to decrease, since more alterations will be found, resulting in more donors rejected by the clinics (Payne *et al*., 2021). A relevant ethical issue comes from the finding of positive results if the screening includes variants with low probability of causing disease and variants of uncertain significance. The last one represents a challenge because there is a lack of information about the correlation between those variants and the corresponding phenotype, as well as the interaction between the genes and the environment (Grosse *et al*., 2010; Yao & Goetzinger, 2016). In addition, there is insufficient observational data to determine penetrance, prevalence and the possibilities of treatment and interventions for the rare diseases found (Grosse *et al*., 2010). In fact, the presence of those variants leads to uncertainty and doubts in interpretation and communication to the individual and, therefore, should be approached with special attention and with the help of clinical geneticists (Lazarin & Haque, 2016; Ontario Health, 2023). Furthermore, it is important to consider that ECS could also introduce additional anxiety for the reasons explained above, and a positive finding will always impose a psychological burden on the donor (Gregg, 2018; Pennings, 2020).

Some authors argue in favor of implementing ECS in screening for gamete donors, as well in individuals who want to conceive, while others confront its use. Payne *et al*. (2021) suggests that a comprehensive ECS panel would be more beneficial for testing oocyte donors but only if followed by matching between donors and recipients. Capalbo *et al*. (2021) also consider beneficial the implementation of ECS in IVF couples and general population. Lastly, the American College of Obstetricians and Gynecologists encourage offering ECS to all women (Urbina *et al*., 2017). Pennings (2020), on the other hand, states that ECS should not be required, especially in the context of oocyte donation. These authors emphasize that there is a high likelihood of finding many alterations with a low probability of being transmitted to the offspring, and that, when balancing the risk reduction that ECS offers and the potential disadvantages, it is extremely hard to justify its mandatory application.

In the context of carrier screening, especially with implemented ECS, when alterations are identified, genetic counseling is essential. This counseling should be performed both pre-test and pos-test if any alterations are detected (Grody *et al*., 2013; Ontario Health, 2023). Initially, it is important that individuals take an informed choice, understand the possibility of encountering pathogenic variants and comprehend their implications on health, as well as the benefits and risks associated, in order to provide an informed consent. Afterwards, if any alteration is found, it is necessary to explain its significance, potential manifestations, penetrance, likelihood of transmission to offspring and the possibility of screening the partner. This counseling should be conducted by clinical geneticists with experience and training in the field (Ontario Health, 2023; Practice Committee of the American Society for Reproductive Medicine and the Practice Committee for the Society for Assisted Reproductive Technology, 2021, Urbina *et al*., 2017).

## CONCLUSION

In conclusion, this study highlights the importance of carrying out a genetic study for gamete donors prior to the donation process, in order to avoid the possible transmission of a genetic alteration to the offspring.
